# Paroxysmal Symptoms in Multiple Sclerosis—A Review of the Literature

**DOI:** 10.3390/jcm9103100

**Published:** 2020-09-25

**Authors:** Joumana Freiha, Naji Riachi, Moussa A. Chalah, Romy Zoghaib, Samar S. Ayache, Rechdi Ahdab

**Affiliations:** 1 Gilbert and Rose Mary Chagoury School of Medicine, Lebanese American University, Byblos 4504, Lebanon; joumana.freiha@lau.edu (J.F.); Naji.riachi@lau.edu.lb (N.R.); Romy.zoghaib@lau.edu (R.Z.); 2 Neurology Department, Lebanese American University Medical Center Rizk Hospital, Beirut 113288, Lebanon; 3 Service de Physiologie-Explorations Fonctionnelles, Hôpital Henri Mondor, Assistance Publique–Hôpitaux de Paris, 51 avenue de Lattre de Tassigny, 94010 Créteil, France; moussachalah@gmail.com (M.A.C.); samarayache@gmail.com (S.S.A.); 4 EA 4391, Excitabilité Nerveuse et Thérapeutique, Université Paris-Est-Créteil, 94010 Créteil, France; 5 Hamidy Medical Center, Tripoli 1300, Lebanon

**Keywords:** multiple sclerosis, paroxysmal symptoms, dyskinesia, ataxia, myokymia, tourettism, paroxysmal weakness, dysesthesia, dystonia, choreoathetosis

## Abstract

Paroxysmal symptoms are well-recognized manifestations of multiple sclerosis (MS). These are characterized by multiple, brief, sudden onset, and stereotyped episodes. They manifest as motor, sensory, visual, brainstem, and autonomic symptoms. When occurring in the setting of an established MS, the diagnosis is relatively straightforward. Conversely, the diagnosis is significantly more challenging when they occur as the initial manifestation of MS. The aim of this review is to summarize the various forms of paroxysmal symptoms reported in MS, with emphasis on the clinical features, radiological findings and treatment options.

## 1. Introduction

Multiple sclerosis (MS) is an autoimmune inflammatory demyelinating disease of the central nervous system (CNS) presenting with a wide range of neurological symptoms. In its relapsing remitting form, it leads to transient episodes of visual, motor, sensory, cerebellar, and autonomic dysfunction lasting for days, weeks, or even months. To be qualifiedas MS relapses, these episodes are required to last more than 24 h. On the other hand, short lasting and highly repetitive paroxysmal events are well recognized complications of MS and are collectively known as paroxysmal symptoms (PS).

PS are characterized by multiple, brief, sudden onset, and stereotyped episodes that could last for seconds to minutes [1,2]. They tend to cluster and could persist for days to months after onset [1,2]. They are considered relapses when occurring over no less than 24 h [3]. These symptoms could appear as the presenting symptoms or during the course of the disease. In the latter setting, PS can be predictive of an MS relapse, its manifestation or its sequelae. PS have a reported incidence of 1.6% to 17% in patients with MS during their disease course of which 24% occur as the initial manifestations of the disease [1,2,4,5,6]. Although PS are classically described in relapsing remitting MS, some types have been also described in the setting of progressive MS [6]. Several clinical subtypes have been characterized and linked to dysfunction of specific anatomical structures within the brain and spinal cord. Although PS are not uncommon, they remain underrecognized and pose a diagnostic challenge, leading to a delay in the initial diagnosis of MS, failure to recognize signs of a relapse in patients with an established MS, and thus deferring initiation of adequate treatments. PS need to be differentiated from other paroxysmal events occurring in the setting of MS such as comorbid migraine, seizures, and sleep disorders. In addition, transient worsening of pre-existing deficits due to extrinsic factors such as fever and fatigue, the so-called Uhthoff’s phenomenon, are not considered PS. Here, we conducted a review of the literature to summarize the different types of PS reported in MS along with their pathogenesis, clinical presentation, magnetic resonance imaging (MRI) findings, and treatment options. In the aim to raise awareness on PS in MS, the broad spectrum of PS will be described in this work.

## 2. Pathogenesis

The pathogenesis of PS remains incompletely understood and several mechanisms have been proposed to explain this peculiar phenomenon. The most popular theory was proposed by Osterman et al. whereby partially demyelinated axons favor the ectopic generation of spontaneous nerve impulses which transversely spread (ephaptic transmission) to other axons within the fiber tracts ultimately manifesting as paroxysmal attacks [7]. This hypothesis is supported by the fact that proprioceptive and tactile stimuli are well established triggers of certain types of PS. Alternatively, the role of “inflammatory irritation” has been proposed based on the favorable response to steroids in many reported cases [8]. Yet, another possible mechanism is ion channel dysfunction in partially demyelinated axons [9]. Such changes would render the axons hypersensitive to minor physiological changes, such as low ionized calcium secondary to hyperventilation, a known trigger of PS. This theory is also supported by the dramatic effect of acetazolamide and anti-epileptics on the frequency of attacks. These theories remain purely speculative and it is not excluded that a combination of the aforementioned factors, and possibly some other unknown phenomena, ultimately result in the generation of PS.

## 3. Paroxysmal Symptoms Categories

PS are a heterogenous group of events that are related to an involvement of motor, sensory, brainstem, and/or cerebellar pathways/centers. Table A1 provides a summary of their features, causative lesions and treatment options.

### 3.1. Motor Symptoms

#### 3.1.1. Paroxysmal Dyskinesia

Paroxysmal dyskinesia is a group of hyperkinetic movement disorders characterized by recurrent episodes of dystonia, chorea, athetosis, or a combination of the latter.

Paroxysmal dystonia (PD) is the 2nd most commonly described movement disorder in MS after tremors [10]. Also known as tonic spasms, these episodes consist of abrupt onset, stereotyped involuntary dystonic posturing. PD can be categorized into painful vs. painless, kinesigenic vs. non-kinesigenic, and generalized vs. focal [10,11,12,13,14]. Generalized dystonia is described as an episode of unilateral upper limb flexion and lower limb extension with a potential to spread to the neck or face [14,15,16]. Focal forms include oromandibular dystonia [17,18], pharyngeal dystonia [19], and hand dystonia (writer’s cramp) [20]. These episodes are often precipitated by movement, hyperventilation, and physical or emotional stressors. They last for less than 1 min [14,15,16,17,18], and tend to gradually increase in frequency over time, reaching up to 100 times per day [15]. Prior to the onset of a dystonic episode, an unpleasant ipsilateral or contralateral sensory aura can occur [4,12,19]. This might be due to subtle muscle contractions insufficient to cause visible movements [21]. Other accompanying features include pathological laughter [22] and autonomic disturbances [23] such as hyperactive bowel sounds, piloerection, and sweating. As previously postulated by Osterman et al. [7], PD can occur with any lesion in the motor pathways. Case reports and series have suggested the involvement of several structures such as the contralateral posterior limb of the internal capsule [24,25,26,27,28], basal ganglia [29,30], thalamus [31,32], brainstem and (cervical) spinal cord, among others [2,22]. Commonly misdiagnosed as focal onset seizures, a normal electroencephalogram (EEG) during the event points towards the non-epileptic origin of these movements.

On the other hand, paroxysmal choreoathetosis (PC) are described as non-rhythmic flowing and twisting movements of one or more limbs. PC share similar characteristics as PD with comparable duration, frequency and precipitating factors [33,34]. The culprit lesion is often located in the mesencephalon and diencephalon [33,34,35].

Paroxysmal dyskinesias are classically treated with pulse steroid therapy alone or in combination with various symptomatic treatments [12,15,16,22,33,34]. In case movements persist despite the use of immunosuppressive treatments alone, symptomatic treatments are recommended. Carbamazepine was reported as one of the most efficacious drugs [36]. Other proposed treatments include acetazolamide [15,37], oxcarbazepine [22], levetiracetam [37], clonazepam [37], and valproic acid [12]. Most of the patients responded within few hours to days of starting the medications and were able to successfully stop them after a month with no residual symptoms.

#### 3.1.2. Myokymia and Hemifacial Spasms

Myokymia is defined as vermicular muscle twitching, most commonly involving the orbicularis oculi muscles [38]. In MS, facial myokymia (FM) may progress into non-sustained contractures (hemifacial spasms, HFS), sustained contractures (paretic hemifacial contracture), or lower motor neuron type facial palsy [39,40]. Alternatively, it can resolve spontaneously [38]. A persistent (more than 6 months) or progressive FM should raise suspicion of secondary causes including MS. Additionally, the pattern of muscle involvement in patients with HFS can differentiate between idiopathic and secondary HSF. Most patients with idiopathic HFS initially have contractions of periocular muscles alone, that later spread to the orbicularis oris and finally the platysma muscle [41]. Conversely, patients with secondary HFS have involvement of the upper and lower facial muscles simultaneously, including the platysma muscle [41]. MRI findings are consistent with ipsilateral lesions in the post-nuclear, postgenu portion of the facial nerve, located in the pons [42]. In a retrospective study of twenty-eight cases of FM in MS patients [43], most of them (twenty-seven patients) had resolution of their symptoms with steroids, gabapentin or carbamazepine.

The latter study also documented treatment outcomes of seven patients with HFS [43]. It is important to highlight that presence of concurrent cranial nerve disorders (i.e., trigeminal neuralgia, glossopharyngeal neuralgia and facial palsy), found in four patients had a major impact on the outcome. Two out of seven patients had spontaneous resolution of symptoms without treatments. Two patients had resolution of HFS symptoms after microvascular decompression or radiofrequency rhizotomy for concurrent trigeminal neuralgia. HFS symptoms also disappeared after microvascular decompression of the facial nerve in one patient. Despite treatments with pregabalin and oxcarbazepine, the last two patients, one of them having glossopharyngeal neuralgia, had good but incomplete response to three-monthly injections of botulinum toxins.

#### 3.1.3. Tourettism

Tourettism is characterized by involuntary motor and phonic tics secondary to neurological and psychiatric disorders [44]. Only a few cases of tics in MS patients have been published. The first patient had a simple phonic tic presenting as paroxysmal throat clearing sounds that were reduced after initiation of pimozide [45]. The second patient with secondary progressive MS was treated with quetiapine for Tourette-like symptoms with vocal tics, stereotyped movements, and coprolalia [44]. The third patient reported decreased vocalization of the phonic tics after administration of dronabinol [46]. All three patients had demyelinating lesions in the basal ganglia and/or thalami [44,45,46].

#### 3.1.4. Paroxysmal Weakness

Paroxysmal weakness (PW) has been reported under several names such as paroxysmal motor symptoms, paroxysmal akinesia and paroxysmal loss of use. PW bears many points of resemblance to other paroxysmal symptoms in the duration, frequency, precipitating factors and treatment options. Patients have described their weakness as “heaviness”, “knee locking”, “leg giving away”, “unexpected falls”, and “objects dropping out of hands” depending on which limbs are involved in the attack [5]. Occasionally, these motor episodes are associated with concomitant symptoms. Examples include blurred vision, dizziness, paresthesia, pathological laughter, and urinary incontinence [47,48,49]. Although these episodes can be initially mistaken for transient ischemic attack, an MRI in favor of MS should suggest otherwise. The appearance of the acute lesion can lend further support to the diagnosis of MS. While acute ischemic lesions demonstrate increased diffusion-weighted imaging (DWI) and reduced apparent diffusion coefficient (ADC) signal, both DWI and ADC are typically increased in acute MS lesions [50]. Nevertheless, very exceptional cases of acute demyelinating lesions might exhibit increased DWI and reduced ADC signal and are therefore indistinguishable from those of acute strokes [50]. In this case scenario, contrast enhancement of the lesion would argue for MS rather than ischemic stroke. It is virtually impossible to correlate the MRI lesions and PW, since all patients had multiple lesions at the time of diagnosis. It can be postulated that any lesion affecting the motor pathway may lead to this presentation. A rare form of generalized PW is cataplexy. In addition to be a symptom of narcolepsy, a comorbid condition with MS, cataplexy has been described as a paroxysmal symptom in MS patients with lesions affecting the hypothalamus or reticular activating system (i.e., within the pons and midbrain) [51]. Treatment with clomipramine was used to control cataplectic attacks in one patient [51].

### 3.2. Sensory Symptoms

Dysesthesia is the generic term used to describe unpleasant cutaneous symptoms such as burning, tingling, anesthesia, itching, tickling, and pain without any primary skin condition [52]. Regardless of the type of dysesthesia, the attacks share similar characteristics. (1) They start and end abruptly, lasting seconds to minutes and occur several times a day. (2) The location can vary, amongst patients, affecting a single dermatome, several dermatomes or involving any body part including the face, extremity, and/or trunk. (3) Attacks can be induced by movements and sensory stimuli. (4) Physical exam of the affected area often reveals sensory disturbances. Dysesthesia involving the trunk or extremities have been linked to lesions affecting the posterior column of the spinal cord supported by attenuation of N20 on somatosensory evoked potentials during a dysesthesia attack [53]. On the other hand, dysesthesia attacks of the head and face are caused by lesions affecting either the origin or the anatomical course of the trigeminal or occipital nerves. Some of the major types of dysesthesia are further discussed below.

#### 3.2.1. Lhermitte’s Sign

Lhermitte’s sign (LS) is a common manifestation of MS occurring in about a third of MS patients, however, it can also be seen in a variety of other conditions involving the spine. LS commonly starts in the early stages of the disease and can be the first clinical manifestation of MS in 10% [54]. It is defined as a short-lasting electric-like sensation felt at the back of the neck radiating to the lower parts of the body following neck flexion and disappearing on resuming normal posture [54]. Most MS patients cope well with this symptom, when problematic, neck braces/collars have been used to decrease neck movements that provoke LS [55]. Carbamazepine had an immediate symptomatic effect in 3 patients with LS [56]. In addition, extracranial picotesla range pulsed electromagnetic fields was found to be effective [55].

#### 3.2.2. Paroxysmal Pruritus

Pruritus has been described in the context of demyelinating diseases of the CNS including neuromyelitis optica spectrum disorders (NMOSD) and MS [57,58]. Paroxysmal pruritus may occur in isolation or can be a predictive symptom immediately before an exacerbation or occur in the recovery phase of a sensory or motor attack [59,60,61,62]. The intense itching often occurs during sleep and follows a specific dermatomal distribution, depending on the location of the lesion in the spinal cord. One MRI was reported with a lesion in the paramedian cervical cord [60]. Carbamazepine was the treatment of choice in all patients with phenytoin, phenobarbital and synacthen used as alternatives [59,60,61,62]. However, it is worth noting that these findings should be interpreted with caution since, at the time of these reports, patients were not tested for the presence of NMOSD-specific antibodies (i.e., anti-aquaporin-4 IgG) since the latter were not known widely [63].

#### 3.2.3. Paroxysmal Pelvic Pain

Paroxysmal pelvic pain is a rare manifestation of MS, but important to note. The episodic attacks are triggered by changes in position and described as lancinating pain radiating to the perineal area. The two described cases did not present solely as MS relapses but rather as sequela of previous spinal attacks [64,65]. Anti-epileptics are the mainstream treatments.

#### 3.2.4. Trigeminal Neuralgia

**Figure A1:**
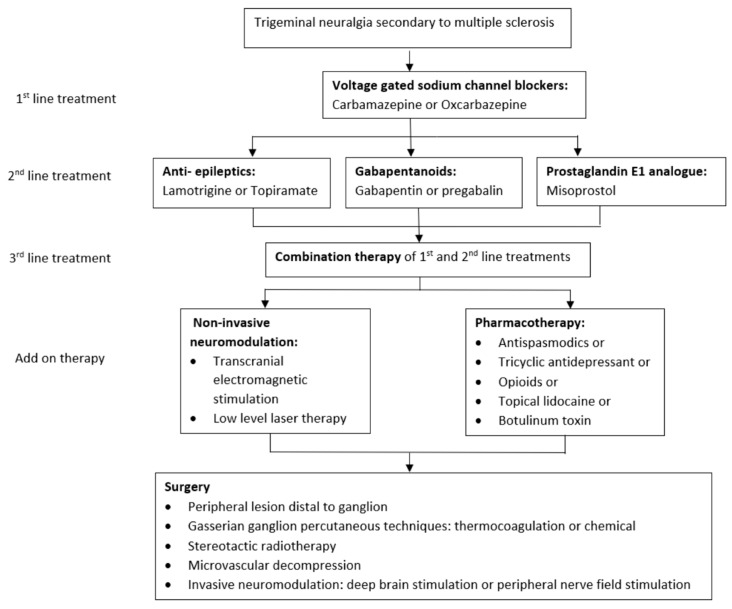
Suggested algorithm for treatment of trigeminal neuralgia secondary to multiple sclerosis.

Trigeminal neuralgia (TN) is characterized by a brief electric shock like pain in one or more divisions of the trigeminal nerve territories. TN is not uncommon in MS with an estimated prevalence of 1.9% to 4.9% [66], with 10–14% being the first manifestation of the disease [67]. Compared to patients with primary TN, TN secondary to MS occurs at a younger age, has a lower frequency of ophthalmic division involvement, higher frequency of bilateral trigeminal nerve involvement [66], and higher incidence of sensory deficit [68]. TN can coexist with a cluster type headache, a syndrome known as cluster-tic syndrome [69]. MS lesions causing TN most commonly involve an area in the ventrolateral pons between the trigeminal root entry zone and the trigeminal nuclei [68]. Treatment options can be categorized into pharmacological, neuromodulation and surgical treatments. Figure A1 provides an algorithm for treatments of TN secondary to MS. The approach to treating TN is mostly inferred from that of primary TN and is based on the use of voltage gated sodium channel blockers, i.e., carbamazepine and oxcarbazepine [70]. Furthermore, there is insufficient evidence to support or refute the effectiveness of other second line medications [70]. Small open-label trials have shown the effectiveness of gabapentin, lamotrigine, topiramate, pregabalin, misoprostol alone or in combination therapies [67,68]. Case reports and retrospective studies showed adequate or partial response to a variety of medications including antispasmodics, antidepressants, opioids, nabiximol, and botulinum toxins [67,71]. Neuromodulation offers an alternative for patients with TN refractory to pharmacotherapy. Options include transcranial electromagnetic stimulation, low level laser and peripheral nerve field stimulation [72,73]. Posterior hypothalamic deep brain stimulation was only found effective in ophthalmic division pain [74]. Patients who have undergone trials of two or three medications at non-toxic doses or those unable to tolerate the side effects should be informed of the availability of surgery. Surgical procedures included peripheral lesions distal to the ganglion, Gasserian ganglion percutaneous techniques (thermocoagulation, chemical lesion with glycerol), stereotactic radiosurgery and microvascular decompression [66,67,68]. A detailed approach to treating TN in the setting of MS can be found in dedicated reviews [68].

#### 3.2.5. Other Types of Neuralgias

Glossopharyngeal neuralgia (GN) is characterized by recurring attacks of severe pain in the back of the throat, radiating to the ear or jaw [66]. These attacks can be associated with episodes of salivation, coughing, hoarseness, and syncope. Triggers include talking and swallowing. Although other disorders causing symptomatic GN have been linked to a lower brainstem injury [66], the symptomatic lesion in patients with MS has not been identified [75].

Occipital neuralgia (ON) is described as a unilateral episodic shooting pain in the distribution of the occipital nerve, mostly in patients with lesions involving the C2-C3 cervical spine [66,76]. GN and ON are treated in the same manner as TN, although evidence to support this approach is lacking.

Seven cases of typical sciatica in MS have been reported. Features differentiating it from sciatica caused by compression of the sciatic nerve include early age of onset, absence of improvement with rest or anti-inflammatory drugs, and absence of mechanical triggers [77].

### 3.3. Brainstem- or Cerebellum-Related Disorders

#### 3.3.1. Autonomic Symptoms

Autonomic dysfunction is a prevalent and significant cause of disability among MS patients. The spectrum of disorders ranges from cardiovascular dysfunction, bladder, bowel and sexual symptoms to thermoregulatory dysfunction. Most MS patients with symptomatic autonomic dysregulation typically have a long history of MS and are significantly disabled (high Expanded Disability Status Scale score). This should be distinguished from paroxysmal autonomic disorders occurring in the setting of an MS relapse which will be discussed in this section.

Cardiac symptoms: Paroxysmal atrial fibrillation (PAF) can rarely occur in the context of an acute brainstem relapse [78,79]. Associated symptoms include vertigo, headache, diplopia, and ataxia. MRI demonstrated multiple areas of demyelination involving the brainstem in the reported cases, presumably affecting the ambiguus and afferent solitary nuclei. PAF episodes were treated with the same medications used to abort cardiac atrial fibrillation.

Orthostatic hypotension in the setting of a brainstem relapse has also been described. The orthostatic drop in blood pressure was of sufficient magnitude to cause recurrent syncope. The culprit lesion was identified in the paramedian tegmentum and base of medulla. Pulse steroids therapy and L-threo-3,4-dihydroxyphenylserine caused regression of the other brainstem signs and reduced syncopal attacks [80].

Urinary incontinence: Patient with MS exhibit diverse manifestations of lower urinary tract dysfunction ranging from detrusor overactivity to detrusor areflexia. These manifestations follow the same natural history of MS course. Urinary symptoms have been reported as paroxysmal, in conjunction with other paroxysmal symptoms. A case of paroxysmal urinary incontinence in conjunction with brainstem symptoms including numbness, dysarthria, unsteadiness of gait was described in a forty-two-year-old woman [81]. These attacks occurred in clusters of two to three times per day, lasting about one hour each, and responded well to carbamazepine. No urinary abnormalities were noted in between the paroxysms. MRI showed a lesion in the right rostral pons most likely affecting the pontine micturition center [81].

#### 3.3.2. Diplopia

Paroxysmal diplopia can be horizontal or vertical in nature depending on the underlying cause. Intermittent horizontal diplopia can be the result of recurrent episodes of bilateral eye adduction coupled with miosis [82,83], a condition known as convergence spasms. These paroxysms are believed to be provoked by irritation of the medial longitudinal fasciculus with lesions affecting the dorsomedial midbrain [83] and pons [82], among others [2]. Intermittent vertical diplopia owing to paroxysmal superior rectus and levator palpebrae spasm was described in the setting of a lesion affecting the third cranial nerve [84]. Alternatively, lesions affecting the third cranial nerve along with neighboring uncrossed corticospinal tracts and horizontal gaze projections can lead to a paroxysmal complex ocular motility disorder and crossed hemiplegia [85]. Treatments with reported benefits include steroids, carbamazepine, bromocriptine, or cycloplegia [82,83,86].

#### 3.3.3. Visual Evoked Nausea and Vomiting

Involvement of the vomiting center and the chemoreceptor trigger zone in the area postrema has been implicated as a cause of paroxysmal nausea and vomiting (PNV) [87,88]. The attacks are triggered by any kind of movement in the patient’s field of vision. A possible explanation for the visual evoked PNV are lesions affecting the efferent connections between the visual cortex and the pontine/medullary reticular formation. Patients with PNV found relief after pulse steroid therapy and after eye closure [87,88].

#### 3.3.4. Vertigo

Central position vertigo (CPV) is a type of position vertigo in which the otolithic displacement is not the cause of the disease. A CPV is suspected when the vertigo is associated with neurological signs, atypical nystagmus pattern, poor response to therapeutic maneuvers (i.e., Epley maneuver) and recurrence on at least 3 different occasions [89]. When a demyelinating lesion is present on MRI, posterior pontine, midline vermis, and middle cerebellar peduncle are mostly affected [90].

#### 3.3.5. Dysarthria and Ataxia

Although paroxysmal ataxia is a well-established symptom of MS, it rarely if ever occurs in isolation. Ataxia is classically described in combination with other symptoms such as dysarthria, crossed paresthesia and ocular flutter. Goodwin and carpenter reported a total of 59 cases of paroxysmal dysarthria and ataxia syndrome (PDA) in their 2016 article [91]. Since then, only one additional case was published [92]. Each episode starts with slurring of speech followed by ataxia of gait [5,91] and/or one or both limbs lasting seconds and recurring several times a day [93,94]. Ipsilateral sensory symptoms including paresthesia, numbness and burning sensation of the face, tongue and upper limb may precede or follow PDA attacks [5,94]. The latter were provoked by hyperventilation, as well as emotional and physical stress. Midbrain lesions, at the level or below the red nucleus, are mostly linked to PDA [92,93,94,95,96]. However, lesions of the dorsolateral pons and cerebellum have been also described [91,97].

Hemiataxia and crossed paresthesia is another paroxysmal syndrome described in MS, albeit much less frequent than PDA, with only three reported cases [7,98]. Abnormal blink reflex responses localize the lesion to the pons [98]. A lesion in the upper part of the pons affecting the brachium conjunctivum, ventral central trigeminal tract and lateral spinothalamic tract may explain these crossed symptoms [7]. Another syndrome is ataxia and ocular flutter presenting as visual blurring. This has been described in cerebellar peduncle lesions [99].

There is no evidence-based approach on how to treat MS patients with paroxysmal ataxia and the effectiveness of various treatments is based on case reports. Standard antiepileptics such as carbamazepine [94,95], levetiracetam [91], lamotrigine [100], lacosamide [92], and phenytoin [98] were found to be effective in aborting ataxic episodes. One reported patient with attacks refractory to steroid and various antiepileptic treatments, achieved full recovery after 1 month of treatment with fingolimod [96]. The resolution of symptoms in this patient could be attributed to the natural history of an MS relapse or to fingolimod ability to limit glutaminergic transmission, therefore, potentially reducing neuronal hyperexcitability [96].

Paroxysmal dysarthria attacks have been mostly described in association with ataxia. However, isolated paroxysmal dysarthria attacks may occur with lower midbrain lesions specifically paramedian lesions below the red nucleus [100,101,102]. Paroxysmal dysarthria consisted of short episodes (few seconds) of inability to speak fluently recurring several times a day, associated with “jaw tension”, “handwriting disturbance” and “facial numbness”. Reported cases were firstly treated with intravenous steroid therapy in combination or followed by carbamazepine. Lamotrigine abolished attacks two weeks after initiation in a case resistant to carbamazepine [100].

#### 3.3.6. Breathing Symptoms

Respiratory complications occur in advanced cases of MS but may also occur earlier in the course of the disease with brainstem relapses. Howard et al. reported five cases with paroxysmal respiratory symptoms related to acute MS exacerbations [103]. Three cases suffered from paroxysmal hyperventilation (PH) episodes accompanied by either facial or upper limb spasms or eyelid fluttering. PH episodes were precipitated by sudden movements or exercise. Two cases reported paroxysmal apneustic breathing episodes described as paroxysms of gasping inspiration, followed by apnea and development of cyanosis and terminating with shallow and rapid breathing. Unlike PH, apneustic episodes were triggered by deep breathing, emotional stress and swallowing. These attacks resolved with remission of the relapse or after adrenocorticotropic hormone treatment in a single case.

#### 3.3.7. Cough

Paroxysmal cough in a young patient with a negative workup for all respiratory and gastrointestinal problems should be investigated for neurological origins of coughs including MS. Other features pointing towards a neurological origin are a preceding aura (tingling in the throat), an abnormal neurological examination and a cough unresponsive to anti-tussive medications [104]. Involvement of the cough center at the medullary level could be responsible for paroxysmal coughs. Anti-epileptic medications improved the tussive crises in the reported patient [104].

#### 3.3.8. Hiccups

Hiccups with or without vomiting were rarely described in MS. Intermittent bouts of hiccups with hiccup-free intervals have been reported [105,106,107,108]. Some constituted the presenting symptom of the disease [105,107], some were associated with other symptoms (e.g., syncope, vomiting, neurological symptoms) [106,107,108] as well as with cervical or brainstem lesions (e.g., ventral paramedian portion of the medulla) [106,107,108]. A malignant form of hiccups was described in association with cervico-medullary lesions leading to quadriparesis and respiratory failure within hours, but the report does not characterize the nature of hiccups (continuous vs. intermittent bouts) [109]. Extrapolating from other diseases, lesions disrupting the corticobulbar tracts and nuclei (nucleus tractus solitarius and nucleus of the vagus nerve) in the brainstem are believed to be responsible for hiccups [109]. Spontaneous resolution or no response to some treatments has been documented in this context. In addition, case reports on intractable hiccups suggest potential benefits of some treatments (intravenous pulse steroids, prednisone combined with metoclopramide, carbamazepine) [105,108,109].

However, as with pruritus, one should keep in mind that NMOSD-specific antibodies were not studied in the recruited patients. In addition, the recent literature showed that intractable hiccups with or without vomiting are a characteristic manifestation of NMOSD, with 17% of NMOSD patients presenting with this symptom during the course of their disease [110]. As mentioned previously, the distinction between NMOSD and MS could not be made prior to the discovery of specific NMOSD-specific antibodies [63], hence the MS cases presenting with hiccups prior to the discovery of these antibodies should be interpreted with caution and formal conclusions cannot be drawn on this matter.

### 3.4. Other Disorders

One important type of symptomatic autonomic dysregulation that worth mentioning is paroxysmal hypothermia, mostly a manifestation of secondary progressive forms of MS [111]. In contrast to “classical” PS, paroxysmal hypothermia episodes are not stereotyped, can last for weeks, and have variable associated symptoms [111,112,113,114,115,116,117,118,119]. Episodes of paroxysmal hypothermia were fatal in some cases. The accompanying clinical features included new or worsening of pre-existing neurological deficits, psychiatric symptoms, confusion, and loss of consciousness. Some abnormalities on laboratory tests were reported such as thrombocytopenia, elevated transaminase levels, and inappropriate antidiuretic hormone levels. Neuroimaging and histopathological studies included in some of these reports suggest the involvement of the hypothalamus (e.g., the preoptic area)—the temperature regulatory center—or possibly its outflow to other structures (i.e., brainstem and spinal cord). The contribution of inflammatory milieu (e.g., proinflammatory cytokines) might also account for the occurrence of these symptoms, especially in patients that did not have evidence of hypothalamic abnormalities.

## 4. Conclusions

PS are relatively common but underrecognized manifestations of MS and are often misdiagnosed as diseases such as epilepsy, transient ischemic attacks and psychosomatic disorders. Much remains unknown about this heterogeneous group of disorders. Treatment paradigms are mostly based on expert opinions and case series rather than high quality supportive evidence. Advances in our understandings of the frequency, impact, and proper management of these symptoms is only possible if these are specifically addressed in large MS cohorts. Until then, awareness among neurologists of these unusual symptoms will help avoid unnecessary diagnostic delays.

## Appendix A

**Table A1:** Summary of paroxysmal symptoms features, causative lesions and treatment options.

Types	Features	Causative Lesions	Treatments
Paroxysmal Motor Symptoms			
**Generalized dystonia** [2,10,22,23,24,25,26,27,28,29,30,31,32]	▪ Unilateral upper limb flexion and lower limb extension ▪ Can spread to neck or face ▪ Preceded by sensory aura ▪ Associated with pathological laughter or autonomic symptoms	▪ Thalamus ▪ Subthalamic nucleus ▪ Basal ganglia ▪ Cerebellum and cerebellar peduncles ▪ Upper ventral pons ▪ Cerebral peduncles ▪ Corpus callosum ▪ Medulla oblongata ▪ Centrum semiovale ▪ Any level of the spinal cord (i.e., cervical)	▪ Pulse steroid therapy alone ▪ Pulse steroids + antiepileptics ^#^ ▪ Antiepileptics ^#^ alone ▪ Acetazolamide ▪ Clonazepam
**Oromandibular dystonia** [17,18]	▪ Mouth opening, deviation of the jaw and lips or tongue protrusion ▪ Can lead to speech and swallowing problems	▪ Genu of the internal capsule	▪ Trihexyphenidyl hydrochloride
**Pharyngeal dystonia** [19]	▪ Transient painful dysphagia ▪ Associated with an abnormal sensation in the throat		▪ Carbamazepine
**Writer’s cramp** [20]	▪ Hand dystonia	▪ C6–C7 cervical spine	
**Choreoathetosis** [10,33,34,35]	▪ Non-rhythmic flowing and twisting movement of one or more limbs	▪ Dorsolateral thalamus ▪ Basal ganglia ▪ Posterior part of the internal capsule ▪ Mesencephalic peduncle	▪ Pulse steroid therapy alone ▪ Antiepileptics ^#^ ▪ Acetazolamide
**Myokymia and hemifacial spasms** [38,39,40,41,42,43]	▪ Vermicular muscle twitching ▪ Hemifacial contractions	▪ Post nuclear, post genu portion of the facial nerve located in the pons	▪ Pulse steroid ▪ Carbamazepine/oxcarbazepine ▪ Gabapentin/pregabalin ▪ Botulinum toxin
**Tourettism** [44,45,46]	▪ Throat clearing sounds ▪ Vocal tics and motor tics ▪ Coprolalia	▪ Basal ganglia ▪ Thalamus	▪ Pimozide ▪ Quetiapine ▪ dronabinol
**Weakness** [5,47,48,49,50]	▪ Unilateral upper or lower limb loss of movement ▪ Associated with dizziness, paresthesia, laughter and urinary incontinence	▪ Same as generalized dystonia	▪ Same as generalized dystonia
**Cataplexy** [51]	▪ Sudden collapse without loss of consciousness	▪ Hypothalamus ▪ Reticular activating system (pons and midbrain)	▪ Clomipramine
**Paroxysmal Sensory Symptoms**			
**Lhermitte’s sign** [54,55,56]	▪ Electric like sensation felt at the back of the neck following neck flexion	▪ Posterior column of the spinal cord	▪ Neck brace/collar ▪ Carbamazepine ▪ Extracranial picotesla range pulsed electromagnetic fields
**Pruritus** [59,60,61,62]	▪ Intense itch with a dermatomal distribution ▪ Often occurs during sleep	▪ Paramedian spinal cord	▪ Carbamazepine ▪ Phenytoin ▪ Phenobarbital ▪ Synacthen
**Pelvic pain** [64,65]	▪ Lancinating pain ▪ Radiating to the perianal area ▪ Triggered by change in position		▪ Antiepileptics ^#^
**Trigeminal neuralgia** [66,67,68,69,70,71,72,73,74]	▪ Shock like pain in one or more divisions of the trigeminal nerve	▪ Ventrolateral pons	▪ Refer to Figure A1
**Occipital neuralgia** [66,76]	▪ Shooting pain in the distribution of the occipital nerve	▪ C2–C3 cervical spine	▪ Same medication as trigeminal neuralgia
**Glossopharyngeal neuralgia** [75]	▪ Severe pain in the back of the throat ▪ Radiating to the ear or jaw ▪ Associated with salivation, coughing, hoarseness and syncope	▪ Lower brainstem *	▪ Same medication as trigeminal neuralgia
**Sciatica** [77]	▪ Pain radiating to the back of the leg		
**Paroxysmal Brainstem- or Cerebellum-Related Symptoms**			
**Atrial fibrillation** [78,79]	▪ Symptoms of atrial fibrillation: Palpitation, presyncope and sweating ▪ Associated with vertigo, headache, diplopia and ataxia	▪ Brainstem affecting the nucleus ambiguus and afferent solitary nuclei *	▪ Medications used to treat cardiac causes of atrial fibrillation
**Orthostatic hypotension** [80]	▪ Syncope upon standing	▪ Paramedian tegmentum and base of medulla	▪ Pulse steroids ▪ L-threo-3,4-dihydroxyphenylserine
**Urinary incontinence** [81]	▪ Episodes of urinary incontinence ▪ Associated with numbness, dysarthria	▪ Rostral pons affecting pontine micturition center	▪ Carbamazepine
**Convergence spasm** [82,83]	▪ Horizontal diplopia ▪ Bilateral eye adduction coupled with miosis	▪ Dorsomedial midbrain affecting medial longitudinal fasciculus ▪ Pons	▪ Pulse steroid ▪ Carbamazepine ▪ Bromocriptine ▪ Cycloplegia
**Superior rectus and levator palpebrae spasm** [84]	▪ Vertical diplopia	▪ Midbrain lesions affecting 3rd nerve fascicle	▪ Carbamazepine
**Ocular motility disorder and crossed hemiplegia** [85]	▪ Diplopia and hemiplegia	▪ Junction of the mesencephalon and pons	▪ Carbamazepine
**Visual evoked nausea and vomiting** [87,88]	▪ Nausea and vomiting triggered by movement in the visual field	▪ Lesions affecting the connection between the visual cortex and reticular formation *	▪ Pulse steroid ▪ Eye closure
**Vertigo** [89,90]	▪ Recurrent positional vertigo ▪ Associated with atypical nystagmus pattern ▪ Poor response of Dix-Hallpike maneuver	▪ Posterior pons ▪ Midline vermis ▪ Middle cerebellar peduncle	
**Hemiataxia and crossed paresthesia** [7,98]	▪ Hemiataxia preceded by contralateral facial paresthesia	▪ Upper part of the pons *	▪ Carbamazepine ▪ Phenytoin
**Ataxia with ocular flutter** [99]	▪ Incoordination of the limbs + gait ataxia + visual blurring	▪ Cerebellar peduncle	▪ Carbamazepine
**Dysarthria and ataxia** [2,91,92,93,94,95,96,97]	▪ Starts with dysarthria followed by gait ataxia ▪ Preceded or followed by ipsilateral sensory symptoms	▪ Midbrain (at the level or below red nucleus) ▪ Dorsolateral pons ▪ Cerebellum	▪ Pulse steroid ▪ Antiepileptics ^#^
**Dysarthria without ataxia** [100,101,102]	▪ Slurred speech ▪ Associated with jaw tension, facial numbness and handwriting difficulties	▪ Midbrain paramedian lesions below the red nucleus	▪ Pulse steroid therapy +/- carbamazepine ▪ Carbamazepine alone ▪ Lamotrigine
**Hyperventilation** [103]	▪ Hyperventilation ▪ Associated with facial +/- upper limb spasms or eyelid flutter	▪ Brainstem	▪ Self-remitting
**Apneustic episodes** [103]	▪ Gasping inspiration followed by apnea and cyanosis and terminating with shallow rapid breathing	▪ Brainstem	▪ Self-remitting
**Cough** [104]	▪ Cough ▪ Preceded by aura (tingling in the throat) ▪ Unresponsive to anti-tussive medication	▪ Medulla affecting the cough center	▪ Antiepileptics ^#^
**Hiccups** [105,106,107,108,109]	▪ Hiccups ▪ vomiting ▪ syncope ▪ neurological symptoms	▪ Lesions disrupting the corticobulbar tracts and nuclei * ▪ Brainstem ▪ Cervical spine	▪ Pulse steroid therapy ▪ Prednisone + Metoclopramide ▪ Carbamazepine
**Other Symptoms**			
**Hypothermia** [111,112,113,114,115,116,117,118,119]	▪ Associated with neurological worsening, neuropsychiatric symptoms, and laboratory findings	▪ Lesions involving the hypothalamus or its outflow	Rewarming

* Hypothesized lesions: Not reported on MRI studies. # Antiepileptics include carbamazepine, oxcarbazepine, phenytoin, valproic acid, levetiracetam, and lacosamide.
